# Septic Emboli to the Brain Secondary to a Patent Foramen Ovale: A Rare Complication of Internal Jugular Vein Catheter

**DOI:** 10.7759/cureus.59419

**Published:** 2024-04-30

**Authors:** Nikhil Verma, Nimish Gupta

**Affiliations:** 1 Oncology, Metro Heart Institute With Multispeciality, Faridabad, IND; 2 Nephrology, Metro Heart Institute With Multispeciality, Faridabad, IND

**Keywords:** hemodialysis, cvc, central venous catheter, ijv catheter, internal jugular vein catheter, pfo, patent foramen ovale, brain, septic emboli

## Abstract

The prolonged use of hemodialysis catheters is associated with several complications with infection being the most common. The increased susceptibility to infections in patients on hemodialysis can be attributed to decreased immunity, though age, other comorbidities, and properties of the catheter act as modifiers. Hematogenous spread of the infection can lead to sepsis and seeding into other organs. In this article, we report an unusual case of septic emboli to the brain in a 30-year-old male on prolonged use of a right internal jugular vein (IJV) catheter for hemodialysis. An interesting finding in the case was the presence of a patent foramen ovale (PFO), a persisting embryonic structure that allows right-to-left shunting. It is suspected that this PFO led to the passage of septic emboli from the right IJV site to the brain. Before our case, septic emboli to the brain have been reported to occur from valvular vegetation in case of infective endocarditis. The mainstay of managing patients with septic emboli is the use of antibiotics; additional interventions may be needed on a case-to-case basis.

## Introduction

Infection is the most common complication of prolonged use of hemodialysis catheters in patients on dialysis. It is one of the major causes of death in such patients [1,2]. The risk is two to three times higher with central venous catheters (CVCs) like internal jugular vein (IJV) catheters than with arteriovenous fistula or vascular grafts [3]. Hematogenous spread of the infection leads to sequelae such as infective endocarditis, arthritis, osteomyelitis, endophthalmitis, abscesses, and septic shock [2,4].

The foramen ovale is an embryonic structure that shunts the blood from the right atrium to the left atrium to avoid the high resistance in the pulmonary circulation during the fetal period. The structure closes after birth owing to the decreased pressure in the pulmonary circulation; however, in 20-35% of the adult population, it may remain patent. The foramen can provide a route for transferring the emboli, similar to a cyanotic heart condition with a right-left shunting or a pulmonary arteriovenous fistula [5]. In both these conditions, the infective emboli bypass the pulmonary microcirculation and can therefore enter the systemic arterial circulation. This can allow seeding of the bacterial emboli in areas of microcirculation of the brain [5,6].

The most common sources of emboli in the brain include the heart and the carotid arteries. Septic emboli can result in infarcts, secondary hemorrhages, abscesses, or mycotic aneurysms [7]. According to our literature search, there have been a handful of reports on septic emboli in the brain, with the majority in association with infective endocarditis [8,9].

Septic emboli to the brain secondary to an IJV catheter in patients on hemodialysis have sparsely been reported. In this article, we provide a unique case of multiple brain septic emboli secondary to a patent foramen ovale (PFO) in a patient with an IJV catheter for hemodialysis.

## Case presentation

A 30-year-old male, a known case of chronic kidney disease stage V on maintenance hemodialysis twice weekly with a non-cuffed temporary catheter IJV in situ for the past two months, presented with altered sensorium and fever for seven days. For this, the patient had been taking meropenem for seven days at home. Before the presentation, he underwent hemodialysis two days back when oozing pus from the right IJV catheter (hemodialysis line) was noted and the line was subsequently removed and a new left IJV catheter was inserted. The rest of his past medical history included hypertension, chronic kidney disease stage V, dilated cardiomyopathy with a left ventricle ejection fraction of 25%, and recurrent pancreatitis and cholecystitis. The patient seemed drowsy and did not respond to verbal commands. Significant general examination findings included a temperature of 101°F. Systemic examination revealed the following significant findings: decreased bilateral air entry into the lungs and bilateral crepitations on lung auscultation. The important investigation findings are listed in Table 1.

**Table 1:** Important investigation findings.

S. no.	Parameter	Value	Reference range
1	Hemoglobin	6.8 g/dL	13.0-17.0 g/dL
2	Total leucocyte count	54,360 cells/mm^3^	4,000-11,000 cells/mm^3^
3	Percentage of polymorphs	90.4%	40-80%
4	Serum procalcitonin	80.2 μg/L	<0.5 μg/L
5	Serum bilirubin (total)	5.6 mg/dL (total)	0.2-1.3 mg/dL
6	Serum bilirubin (direct)	5.5 mg/dL	0.00-0.4 mg/dL
7	Serum alkaline phosphatase	396 U/L	38-126 U/L
8	Serum amylase	45 U/L	30-110 U/L
9	Serum lipase	149 U/L	23-300 U/L
10	Serum urea	78 mg/dL	19.2-42.8 mg/dL
11	Serum creatinine	4.9 mg/dL	0.66-1.25 mg/dL
12	Serum sodium	137 mmol/L	135-155 mmol/L
13	Serum potassium	5.1 mmol/L	3.5-5.5 mmol/L
14	Serum calcium (total)	10.7 mg/dL	8.4-10.2 mg/dL
15	Serum parathyroid hormone (intact)	16.2 pg/mL	9.2-44.6 pg/mL
16	Random blood sugar	163 mg/dL	<200 mg/dL

**Figure 1:**
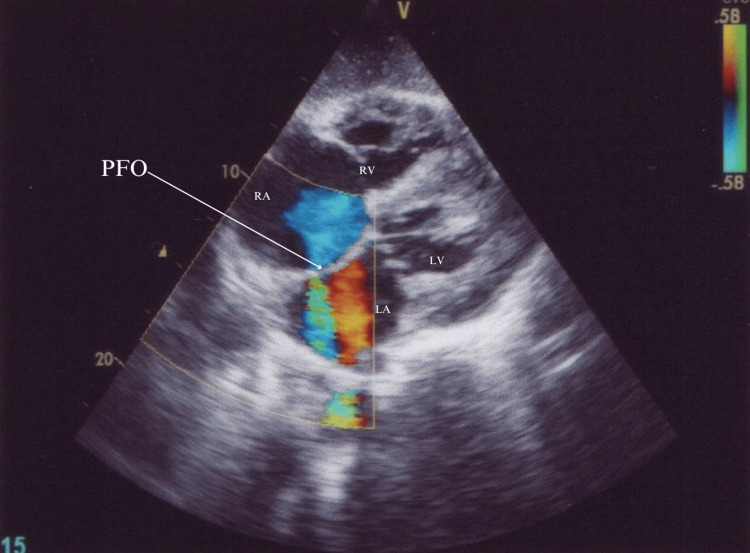
Echocardiogram suggesting the presence of a patent foramen ovale (PFO). RA: right atrium; RV: right ventricle; LA: left atrium; LV: left ventricle

**Figure 2:**
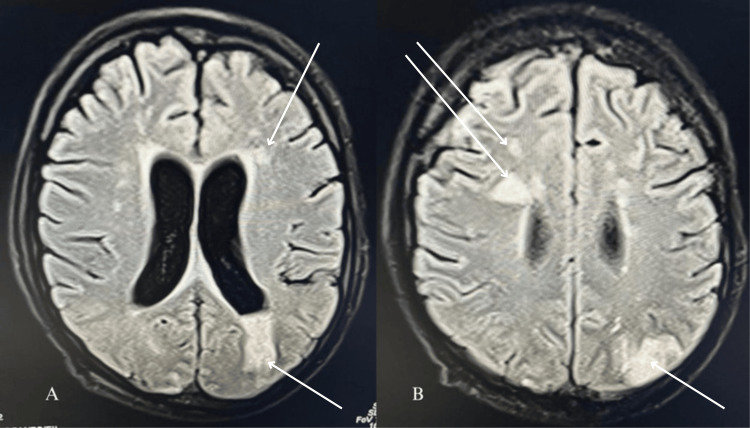
MRI flair sequence showing multiple septic emboli in the brain.

An echocardiogram suggested moderate tricuspid regurgitation, moderate pulmonary hypertension, no valvular vegetation, and the possibility of a tiny PFO (Figure 1). Given the suspected lung infection and catheter-related bloodstream infection, the patient received broad-spectrum antibiotics (meropenem and teicoplanin) and other supportive care. Because of the low Glasgow Coma Scale (GCS) score E1V1M2, the patient was intubated. Chest X-ray showed bilateral infiltrates suggestive of fluid overload. Blood and urine cultures yielded no growth after 48 hours of incubation at 37°Celcius. Magnetic resonance imaging (MRI) brain was done which revealed septic emboli (Figures 2A, 2B).

A repeat echocardiogram confirmed a PFO of a diameter of around 4 mm. Given a prior infection at the hemodialysis line, PFO was suspected to be the cause of the brain septic emboli, which could be the cause of his altered sensorium. Despite management with antibiotics, the patient succumbed.

## Discussion

Septic emboli to the brain is an uncommon phenomenon with the majority occurring secondary to infective endocarditis. Previously, Leso et al. reported a case of septic brain emboli with tricuspid valve endocarditis as well as a PFO in a 34-year-old male [9]. Liang et al.'s case report discussed brain septic emboli in a 51-year-old male associated with infective endocarditis but not a PFO while Yoon et al.'s study described septic emboli in the brain in a 39-year-old male without PFO or infective endocarditis [8, 10]. Our case is probably the first report of septic emboli secondary to an IJV catheter and a PFO (without infective endocarditis).

Patients on hemodialysis with CVCs are predisposed to infections and, therefore, sepsis due to the following factors: firstly, patients with chronic kidney disease have decreased glomerular filtration, and as a result, urea assimilates. The urea of gut-bacteria origin also accumulates and promotes disintegration of the gut barrier as well as stimulates white blood cells. Further, methylglyoxal, a uremic toxin, promotes oxidative bursts within polymorphonuclear cells. Next, both cell-mediated and humoral immunity are impaired in these patients [1,11]. Secondly, age, diabetes mellitus, malnutrition, site of access to CVC, duration of use of the CVC, manipulation of the CVC, colonization of the CVC, and colonization of the skin and nasal flora are among other factors [1-3].

The pathophysiology of infection of CVCs includes the following steps: migration of microorganisms from the skin to the external surface of the catheter, from the external surface to the lumen, septicemia, and finally inoculation in different organs of the body. Bacteria with the properties of adherence and biofilm formation are efficient in colonizing the catheter. Notable examples include *Staphylococcus aureus* which binds to the host's fibronectin and coagulase-negative staphylococci which attach to the polymers [1]. Septic emboli in the brain can present with fever, headache, focal neurologic deficits, or non-specific symptoms [8-10].

According to our literature search, different causative organisms were isolated in different cases of septic emboli in the brain. In Leso et al.'s report, the responsible agent was *Klebsiella oxytoca* [9]. Yoon et al. isolated *Gardnerella vaginalis* from the blood of their patient [10]. On the other hand, the causative agent for brain septic emboli in Liang et al.'s case report was *Haemophilus parainfluenzae* [8]. In our case, the blood cultures were sterile.

The patient in Yoon et al.'s case report of *Gardnerella vaginalis*-induced septic emboli recovered with intravenous metronidazole, intravenous ceftriaxone, and oral erythromycin [10]. Liang et al. used ceftriaxone to treat* Haemophilus parainfluenzae*-induced septic emboli and the patient recovered with no focal neurologic deficits. However, the patient required interventions for the valvular conditions [8]. The patient reported by Leso et al. received parenteral antibiotics and underwent debulking of the tricuspid valve. Additionally, three applications of NobleStich were done to close the PFO but it reappeared in a follow-up study. He lost the majority of his right eye vision and declined further intervention [9]. According to Zakhari et al., interventions may be required to treat or prevent complications of brain emboli [7]. In our report, the patient succumbed despite the use of antibiotics.

Some patients with brain septic emboli like our patient, could ultimately be reported to have a PFO, like the patient in this report. In case of a patient with no prior echocardiogram finding suggestive of a PFO, a transesophageal echocardiogram (TEE) (or sometimes transthoracic echocardiogram) with bubble study should be performed.

## Conclusions

To conclude, brain septic emboli should be considered in a patient with an IJV catheter and a history of altered sensorium, focal neurologic deficits, headache, or other non-specific symptoms. PFO, which is seen in 20-35% of the adult population, may mediate the embolization of the septic emboli and should be investigated. It can be diagnosed with a TEE with a bubble study. The management should involve antibiotics and additional interventions on a case-to-case basis.
